# ADAM-15 Disintegrin-Like Domain Structure and Function

**DOI:** 10.3390/toxins2102411

**Published:** 2010-10-19

**Authors:** Dong Lu, Mike Scully, Vijay Kakkar, Xinjie Lu

**Affiliations:** 1 Thrombosis Research Institute, Manresa Road, London, SW3 6LR, UK; Email: dl3@sanger.ac.uk (D.L.); mscully@tri-london.ac.uk (M.S.); president@tri-kakkar.ch (V.K.); 2 Sanger Institute, Wellcome Trust Genome Campus, Hinxton, Cambridge, CB10 1SA, UK

**Keywords:** ADAM protein, Snake venom toxin, disintegrin, integrin, RGD-motif

## Abstract

The ADAM (a disintegrin-like and metalloproteinase) proteins are a family of transmembrane cell-surface proteins with important functions in adhesion and proteolytic processing in all animals. Human ADAM-15 is the only member of the ADAM family with the integrin binding motif Arg-Gly-Asp (RGD) in its disintegrin-like domain. This motif is also found in most snake venom disintegrins and other disintegrin-like proteins. This unique RGD motif within ADAM-15 serves as an integrin ligand binding site, through which it plays a pivotal role in interacting with integrin receptors, a large family of heterodimeric transmembrane glycoproteins. This manuscript will present a review of the RGD-containing disintegrin-like domain structures and the structural features responsible for their activity as antagonists of integrin function in relation to the canonical RGD template.

## 1. Introduction

The “disintegrin” terminology was initially applied in 1990 to describe a family of cysteine-rich, RGD-containing proteins, isolated from the venom of snakes that inhibit platelet aggregation and integrin-mediated cell adhesion [1,2,3]. Subsequently, homologous proteins in which the arginine residue was replaced within the RGD motif including the motifs: KGD [4,5], MGD [6], VGD [7], WGD or MLDG [8,9] were also adopted into the disintegrin family. The RGD sequence is also found in proteins such as decorsin [10] and ornatin [11] from leech toxins, and variabilin [12] from hard tick toxin. The term “disintegrins” was eventually reserved for a particular form of snake venom toxins, and the term “disintegrin-like protein” for RGD proteins with similar properties but different general structures, including the disintegrin-like/cysteine-rich (D/C) domains of the PIII class snake venom metalloproteinases (SVMP) [13,14]; the ADAM (a disintegrin-like and metalloproteinase) [15,16,17,18] and ADAMTS (ADAM with thrombospondin motifs) [19,20]; ADAMTSL (ADAMTS-like) families [21] and MDC (metalloproteinase disintegrin-like cysteine-rich) proteins [22,23]. The primary sequences of disintegrin-like domains in the ADAMs family were homologous to those found in snake venom disintegrins. These proteins constitute one subfamily of the so-called adamalysins, which is a protein family belonging to metzincin superfamily of metalloproteinases. Members of this large and conserved protein family have been isolated from a variety of organisms, including mammals, reptiles and invertebrates. Of the 34 ADAM proteins described including the 19 human ADAMs, human ADAM-15 (also called MDC-15, ADAM metallopeptidase domain 15 or metargidin) is the only ADAM protein with the RGD integrin ligand consensus motif in a position analogous to that found in snake venom disintegrins. The RGD sequence is followed by an additional cysteine residue that is not present in RGD-type snake venom disintegrins and has only been detected in non-RGD-type SVMP and ADAM proteins. In this review, we focus on the disintegrin-like domain in ADAM 15 and its structure and function.

## 2. Overview of the ADAM-15 Gene Structure

Human ADAM-15 was discovered in a screen for novel ADAMs by PCR [24]. Expression of a disintegrin-like protein had already been observed in cultured human vascular cells and *in vivo* [25]. ADAM-15 was named metargidin since it carried an RGD sequence in a similar position as snake venom disintegrins (metalloproteinase-RGD-disintegrin protein) [26]. 

**Figure 1:**
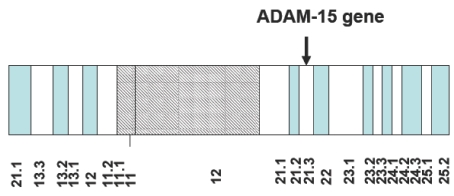
Schematic of partial chromosome 1 with ADAM-15 gene indicated by an arrow.

Human ADAM 15 is located at 1q21.3 of chromosome 1, the largest human chromosome, with ~8% of all human genetic information starting at 153,290,386 bp and ending at 153,301,876 bp from the pter (phosphotriesterase related) and reported to have six transcripts and 11,491 bases. Large introns (intron 1, 1183 bp) occur on the 5¢ and 3¢ sides of the gene with a cluster of exons between them (Figure 1) [27]. 

The gene for ADAM-15 contains 23 exons varying in size from 63–316 bp and 22 introns ranging between 79–1283 bp [28]. The ADAM-15 protein isoforms deduced have combinations of cytosolic regulatory protein interacting motifs with one or both of the almost identical proline-rich regions encoded by exons 20 and 21, where the residues RxLPxxP are indispensable for nephrocystin SH3 binding [29]. 

**Figure 2:**
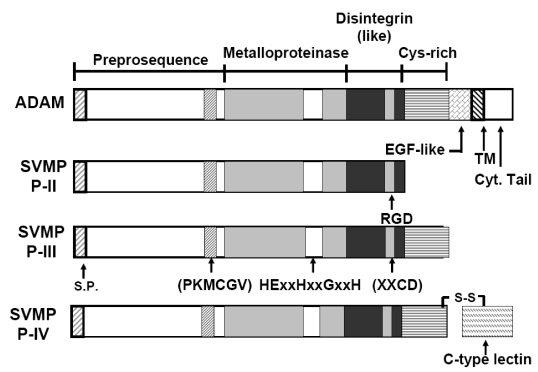
Domain structures of ADAMs compared to snake venom metalloproteinases (SVMP). Members of the ADAM gene family are classified as membrane-anchored ADAMs containing cysteine-rich domain, cytosolic tail, disintegrin-like domain, epidermal growth factor-like domain, metalloproteinase domain, Pro-peptide domain and transmembrane (TM) domain. SVMP can be classified into four subgroups ((P-I to P-IV). S.P. denotes signal peptide.

Human ADAM-15 contains a signal peptide sequence (1 to 17 amino acids (aa)) (Figure 2), followed by a pro‑peptide or pro-domain (18–206 aa) thought to function as an intramolecular chaperone (IMC). The pro-domain is cleaved from the metalloproteinase domain by furin [30], a membrane associated endoprotease that cleaves precursor proteins on the C-terminal side of the consensus sequence. 

## 3. Overview of the Structural Domains of ADAM 15

The pro-domain maintains the metalloproteinase site of ADAM in an inactive state through a cysteine switch [31] similar to that of matrix metalloproteinases (MMPs) and other reprolysins. In this regard, ADAMs, including ADAM-15, are reprolysin-like proteins. The MMPs or matrixins are synthesized as zymogens, which in the case of soluble matrixins are secreted while other members of the family remain bound to the cell surface. The cysteine switch motif in ADAMs may play a role during ADAM biosynthesis. The cysteine residue preferentially coordinates the active site zinc atom sequestering the metalloproteinase domain in an inactive conformation. There are several zinc interacting sites in ADAM-15 (179, 348, 352, 358 aa). Inhibitors of the early secretory pathway block the processing of ADAM-15 and ADAM-9, thus positioning the location of ADAMs processing and activation at the trans-Golgi network [32]. The secondary function of the pro-domain is to chaperone proper folding of the ADAMs, especially the metalloproteinase domain since an ADAM-10 construct lacking the pro‑domain is catalytically inactive *in vivo* [33].

The metalloproteinase domain of ADAM-15 (207–419 aa) and other ADAMs are well conserved, but only 25 out of 40 ADAMs, including ADAM-15 (348–359 aa) and ADAMs 1, 8–10, 12, 13, 16, 17, 19–21, 24–26, 28, 30, 33–40, have the zinc binding catalytic site consensus sequence HExxHxxGxxHD where x is any amino acid. Three His residues and a water molecule tetrahedrally coordinate the zinc, and the Glu residue acts as a catalytic base [34]. 

The disintegrin-like domain is downstream of the metalloproteinase domain. The ADAM-15 disintegrin-like domain contains 90 aa (Met^420^ to Glu^510^), while in other ADAMs this domain contains 60–90 aa. ADAM-15 and has 15 Cys residues showing sequence similarity to the snake venom disintegrins [35].

The cysteine-rich domain of ADAM-15 (511–656 aa) is thought to regulate cell fusion and may be involved in the activation of latent ADAM-15 and removal of the pro-domain through mechanisms that are not fully elucidated [17,36].

An EGF-like domain (657–685 aa) is downstream of the cysteine-rich domain, named for its similarity to epidermal growth factor (EGF) and other related growth factors and containing six, highly conserved cysteine residues with characteristic spacing [37]. Certain data suggest the EGF-like domain is involved in substrate specificity including substrate cleavage and recognition [38].

ADAM-15 and many others are type I membrane proteins anchored to the surface of the cell through the extracellular domain (207–696 aa), TM domain (697–717 aa) including a putative phosphorylation site (715 aa) and cytoplasmic domain near the C-terminus (718–814 aa). The cytoplasmic domain of ADAM-15 interacts with endophilin I and the sorting nexin 9. In contrast, all the ADAMTSs lack a TM domain and are secreted proteases. 

The cytosolic portion (cytoplasmic tail) of ADAM-15 (718–814 aa) and many other ADAMs vary in length (between 40–250 aa) and sequence composition. Similar to other proteolytically active ADAMs, the cytosolic part of ADAM-15 is rich in proline-rich consensus binding sites motif (766–772 aa and 801–806 aa). The cytosolic domain of ADAM-15 is encoded by exons used alternatively in normal tissues giving rise to splice variants with different compositions of putative protein binding motifs [29]. Certain ADAM-15 variants have been associated with poor survival of breast cancer patients [39].

ADAM-15 has another putative phosphorylation site (tyrosine 735 aa) as do many other ADAMs for serine-threonine and/or tyrosine kinases. Phosphorylation of ADAMs may serve to modulate adaptor functions of the protein to assemble complexes of proteins at sites of functional activity. 

## 4. Integrin Interactions of the Disintegrin-Like Domain of ADAM-15

The initial identification of disintegrin-like domains within mammalian ADAMs led to the hypothesis that these regions interact with integrins similar to the related domains in snake venom proteins [17]. There is now considerable evidence that the extracellular domains of ADAMs interact with integrins. Recombinant disintegrin-like domains have been identified with a consensus-binding motif, CRxxxxxCDxxExC, in their disintegrin loops [40]. These interactions influence cell adhesion and cell–cell interactions including those dependent upon the integrins: α_2_β_1_, α_IIb_β_3, _α_4_β_1_, α_4_β_7_, α_5_β_1_, α_6_β_1_, α_6_β_4_, α_9_β_1_, α_V_β_3_ and α_V_β5 [41,42].

## 5. Structural Model of the Disintegrin-Like Domains of ADAMs Proteins

The disintegrin-like domain (D-domain), which is located downstream of the metalloprotease domain, consists of 60 to 90 aa with 6 to 15 Cys residues. Most D-domains of ADAMs have an XCD motif with the exception of ADAM-15, which contains the RGD sequence (484–486) [43] similar to the snake venom disintegrins. Snake venom disintegrins are known to be potent inhibitors of various integrins. Snake venom disintegrins usually have a RGD motif that confers the ability to interact with integrins [44]. The disulfide bridge of RGD-containing disintegrins has been evaluated by chemical methods, NMR spectroscopy and crystallography. The most striking feature is the consistency of the disulfide bonds around the RGD sequence leading to the proposal of an “RGD-containing loop” in each protein, which may be important to their potency and selectivity. NMR studies of this loop in snake venom proteins, including kistrin [45,46,47], flavoridin [48], echistatin [49,50,51], albolabrin [52] and dendroaspin [53] along with the crystal structure of trimestatin [54], show that the RGD sequence is presented at the apex of a β-turn. Although the active sequence in most disintegrins is the RGD tripeptide, some members of the family contain other sequences such as KGD, MVD, MLD, VGD, ECD, or MDG (single letter amino acid code) in complimentary positions and have been characterized as integrin-binding motifs [55]. The disintegrin-like domains of ADAMs and the P-III group SVMPs is larger than the RGD-disintegrins, and most of them have an XCD motif (where X is any amino acid) in their disintegrin-like domains, with the exception of ADAM-15 which contains the RGD sequence [56]. P-III group SVMPs comprising the metalloproteinase, disintegrin-like and cysteine‑rich domains belong to the ADAM/adamalysin/reprolysin family [14,57,58,59,60,61]. Several ADAMs share a sequence Rx_6_DLPE in the D-domain, which can bind avidly to α_9_β_1_ e.g., ADAM-1, -2, -12, and -15, whereas ADAM-10 and -17 do not since they lack this motif [56].

In the study of metalloproteinase domain-containing proteins including the active sequence in terms of structure and function, the crystal structure of the entire ectodomain of mature ADAM‑22 [62] reported following the crystallographic studies of two PIII SVMP proteins, VAP1 (vascular apoptosis-inducing protein-1) and VAP2B (vascular apoptosis-inducing protein 2B), proved very useful [63,64]. Overlaying the SVMP structures on ADAM-22 revealed a positional shift in the D-domain and C-domains (the cysteine-rich domain, 530–676 aa) in ADAM-22 relative to the corresponding domains in the SVMPs.

**Figure 3:**
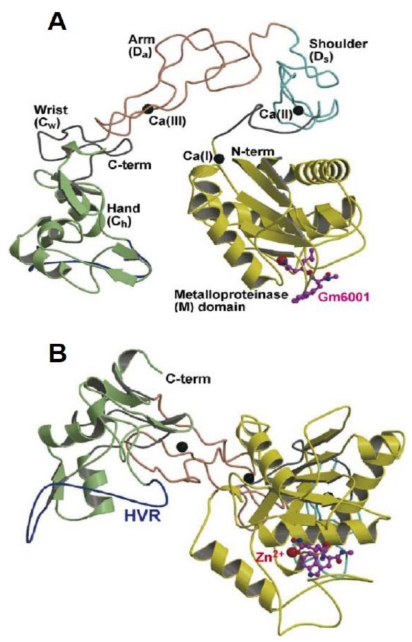
Schematic presentations of the MDC domain. (**A**) and (**B**) present orthogonal views of the MDC domain of catrocollastatin/VAP2B. The M-domain, linker, Ds, Da, Cw and Ch segments, Zn^2+^ binding site, and the HVR are shown in yellow, gray, cyan, pink, gray, light green, red and blue, respectively. The GM6001 (an inhibitor) bound to the protein molecule is shown in ball and stick representation and three Ca binding sites are indicated as I-III, adapted with permission [66].

The D-domain that follows the metalloproteinase domain (M-domain) is seen in VAP1 and VAP2B and divided into 2 sub-domains, the “D-shoulder” (Ds) and ‘‘D-arm” (Da) (Figure 3). Both the Ds (residues 396–440) and Da subdomains (residues 441–487) contain calcium-binding sites [64,65,66]. ADAM-22 contains three putative calcium ions, two in the D-domain and one in the M-domain M, metalloproteinase-like domain (residues 233–435). The Ds- and Da subdomains consist of a series of turns and two short regions of antiparallel β-sheet forming a continuous C-shaped structure, which, along with the N-terminal region of the C-domain, forms a “C-wrist” (Cw) segment. The Cw segment is followed by a ‘‘C-hand” (Ch) segment with a hypervariable region (HVR) at its distal portion [65]. These structural features are summarized in the schematic shown in Figure 3. There are three disulfide bonds in the Ds-segment, three in the Da-segment and one in the Cw-segment, and the segments are connected by single disulfide bonds (Figure 3). X-ray studies of atragin, a protein of P-III family of SVMPs, showed one disulfide bond connecting Ds and Da and another disulfide bond connecting Da and Cw comprised of one cystine residue in the disintegrin-like loop (XXCD) that caused it to become inaccessible for integrin-binding as in VAP1 and VAP2 [61].

## 6. Disintegrin-Like Domain of ADAM-15: Structure and Function

ADAM-15 has been implicated in cell-cell, cell-matrix interactions and in the proteolysis of molecules on the cell surface or the extracellular matrix [67,68,69,70]. The function of ADAM-15 in cell-cell adhesion has been attributed to the D-domain as integrin ligand [71]. Human ADAM-15 provided interesting insights into analyzing the structure/function of the RGD motif dependent interaction with integrin α_V_β_3_ compared to its RGD-independent association with α_9_β_1_ [59]. The study mapped the α_9_β_1_-interaction site to a motif RxxxxxxDLPEF (481–492 aa in human ADAM-15 wherein the RGD motif is at 484–486 aa), that is conserved in all ADAMs excepting ADAM-10 and -17 [59].

## 7. Investigation of the Integrin Interaction Using a Recombinant Disintegrin Domain from ADAM‑15 (ddADAM-15) and Various Mutants

**Figure 4:**
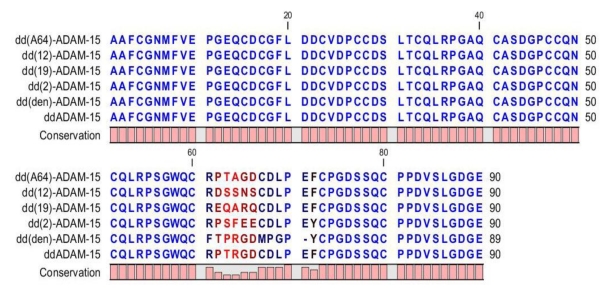
Mutants of disintegrin-like domain of ADAM-15. Sequence alignment of ddADAM-15 and its mutants plotted using CLC protein workbench version 5.2. Numbering is based on the amino acid sequence of ddADAM-15. The dd(A64)-ADAM-15 shows that the R residue in R^64^GD of ddADAM-15 was replaced by Alanine; dd(12)-ADAM-15 denotes that the disintegrin-like RGD-loop of ADAM-15 was replaced by that of ADAM-12. A similar designation was applied to others. dd(den)-ADAM-15 denotes that the disintegrin-like RGD-loop of ADAM-15 was replaced by that of dendroaspin (den), a disintegrin-like protein [53].

The integrin, α_9_β_1_, is widely expressed on smooth muscle and epithelial cells, and mediates adhesion to the extracellular matrix proteins, osteopontin and tenascin-C [72]. We have studied a number of mutants of ddADAM-15 (Figure 4). Recombinant GST-ddADAM-15 and its mutants supported the adhesion of α_9_β_1_-transfected CHO cells, which were shown to reach 50% of the maximum number of adherent cells as dd(den)-ADAM-15 > ddADAM-15 > dd(2)-ADAM-15 > dd(12)-ADAM-15 > dd(19)-ADAM-15 > dd(A64)-ADAM-15 [73]. RGD-independent binding of integrin α_9_β_1_ to ddADAM-15 mediates cell-cell interactions [71]. 

Inhibition of adhesion of α_V_β_3_ integrin-mediated A375-SM cells (a highly metastatic variant of A375 cells, containing α_V_β_3_ integrin) to fibrinogen, by dd-ADAM-15 and its mutants, is ranked as dd(den)-ADAM-15 > dd(2)-ADAM-15 > ddADAM-15 > dd(12)-ADAM-15 > dd(19)-ADAM-15 > dd(A64)-ADAM-15 [73]. Charrier *et al.* have reported that overexpression of ADAM-15, containing a mutation in the RGD motif in Caco2-BBE (human intestinal cell line) monolayers, decreases Jurkat cell adhesion, and showing ADAM-15-mediated binding of T cells on intestinal epithelial cells is RGD sequence-dependent [74]. This suggests that the α_V_β_3_ and α_5_β_1 _integrins expressed on T lymphocyte membranes are putative binding partners for epithelial ADAM-15. *In vitro* experiments confirmed that ddADAM-15 interacts with α_V_β_3_ and α_5_β_1 _integrins on hematopoietic cells [74], and ADAM-15 interaction with these integrins is RGD-dependent [75]. Mosnier *et al.* reported that ADAM-15 is upregulated in epi- and endothelial cells in close contact with α_5_β_1_-expressing leukocytes, suggesting a role in leukocyte migration [76]. ADAM-15 interacts with α_V_β_3_ and α_5_β_1 _integrins, both of which are involved in endothelial cell migration indicating a possible role in atherosclerosis [77,78].

ddADAM-15 is reported to bind via α_2_β_1_ to HT1080 (a human fibrosarcoma cell line), but the binding is weaker than with dd(den)-ADAM-15, which showed the highest binding ability for HT1080 cells [73). The order of binding potency for dd recombinant proteins to α_4_β_1_-mediated MOLT 4 (human acute lymphoblastic leukemia cell line containing α_4_β_1_) cell is demonstrated to be dd(2)-ADAM-15 > dd(19)-ADAM-15 > dd(den)-ADAM-15 = ddADAM-15 > dd(12)-ADAM-15 while dd(A64)-ddADAM-15 showed little/no ability to support cell adhesion compared to its wild-type counterpart. These results suggest that the RGD tripeptide motif may play a role in this binding as dd(A64)-ddADAM-15 failed to bind to this cell line. However, since both dd(den)-ADAM-15 and ddADAM-15 contain the RGD-motif and others do not have the RGD, the difference in potencies emphasizes the importance of the flanking residues in determining potency [73]. 

Although no inhibition to platelet aggregation was found for dd(2)-ADAM-15, dd(A64)-ADAM-15, dd(19)-ADAM-15 and dd(12)-ADAM-15 with ADP-induced platelet aggregation in platelet-rich plasma, ddADAM-15 showed low activity with a maximum 25% inhibition at 10 μM despite the presence of the RGD motif, which is known to be favored for binding to platelet α_IIB_β_3_[73]. Further, yeast-expressed ddADAM-15 inhibited binding of α_IIB_β_3_ to its biological ligands fibrinogen in a dose‑dependent manner. Mutation of the three residues proximal to the RGD tripeptide sequence, RPTRGD sequence to NWKRGD (named NWK mutant), increased its affinity for α_IIB_β_3_. The NWK mutant had a greater inhibitory action on human platelet aggregation than ddADAM-15 [79], suggesting that flanking amino acid residues are important for activity of the RGD motif. 

We have shown that ddADAM-15 can bind to airway smooth muscle cells (ASMCs) and this binding can be modulated by putative disintegrin-like loops within the ddADAM-15 scaffold [80]. This adhesion was mediated by the β_1_-asociated integrins including α_4_β_1_, α_5_β_1_, α_9_β_1_. Hence, ddADAM-15 can serve as a β_1_ integrin antagonist as seen by the inhibition of ASMC binding to fibrinogen. ddADAM-15 inhibited PDGF-induced cell migration with the RGD-motif playing a crucial role as shown by the replacement of the putative disintegrin-like loop with those of ADAM-2, -12 and -19. We established that fibrinogen, rather than fibronectin, binding was blocked by ddADAM-15 in a dose-dependent manner in β_1_-mediated cell binding, implying that ddADAM-15 and fibrinogen share a similar β_1_ integrin binding site. Such a region may not be involved in fibronectin binding despite the location of an RGD sequence in the tenth type III repeat of fibronectin, which is the major binding sitefor β_1_ integrin with α_5_β_1_ [81,82]. The role of RGD in ddADAM-15 may be limited, as this is the only ADAM family protein containing this sequence. It cannot be ruled out that regions beyond the disintegrin-like loop also play a role in integrin-binding since ddADAM-15 and ddADAM-12, which lacks the RGD-motif can interact with β_1_-associated α_9_ integrin [68], and -associated α5 integrin [67] in other cell types.

## 8. ADAM-15 Is Associated with Diseases

The role of ADAM-15 in diseases appears to involve mechanisms as diverse as cell–cell interactions, cell-extracellular matrix (ECM) interactions and shedding activity. There is growing evidence of links between ADAM-15 and human diseases including cancer and atherosclerosis. It was reported that mRNA and/or protein levels of ADAM-15 are upregulated in multiple adenocarcinomas including cancer of the breast, stomach, lung, pancreas and prostate [83]. Horiuchi *et al*. reported that a deficiency of ADAM-15 in a mouse model for retinopathy resulted in reduced neovascularization [84]. Consistently, smaller tumors were formed in the ADAM-15-deficient mice after injection with melanoma cells [85]. Yamada *et al.* demonstrated that pancreatic cancer cells expressed significantly higher levels ADAM-15 mRNA than normal pancreatic epithelial cells [85]. Najy *et al*. [86] found that downregulation of ADAM-15 in the prostate cancer cell line, PC3 decreased migration and adhesion to specific extracellular matrix proteins. Using breast cancer cell lines, the same authors reported that ADAM-15 cleaved cadherin E after growth factor deprivation [87]. The cleaved cadherin E bound and transactivated HER2/HER3, resulting in increased migration and proliferation. Thus, enhanced HER2/HER3 signaling is a potential mechanism by which ADAM-15 could contribute to cancer progression. Sun *et al*. recently reported that ADAM-15 regulates endothelial permeability, which is considered as one of the key cellular processes in the development of inflammatory disorders, including atherosclerosis [88,89], diabetic complications [90] and inflammatory bowel disease [76]. In addition to RGD motif which has an ability to disturb integrin-mediated attachment on the cell surface, the RGD peptides are incorporated into cytoplasm and induce apoptosis [91]. Collectively, several RGD-containing proteins from venom toxins induced apoptosis, such as contortrostatin [92], rhodostomin [93] and salmosin [94]. Since these RGD peptides and RGD-containing proteins interact with integrins, the integrins may serve as targets for anti-cancer agents designed using RGD as a template. Several studies have shown the potential for these RGD proteins to function as integrin antagonists as well as antiagiogenic, antimetastatic and antithrombotic compounds leading to drug development for therapeutic usage [95,96,97].

## 9. Concluding Remarks

The ddADAM-15 selectively modulates integrin-mediated cell adhesion and ASMC migration. The amino acid sequence in the putative disintegrin-like loop plays a crucial role in controlling the selectivity and specificity of the ADAM proteins in their interaction with particular integrins. The RGD-tripeptide in the putative disintegrin-like loop in ADAM-15 serves as an integrin recognition sequence since conversion of RGD into AGD reduced potency, inhibiting A375-SM cell adhesion to fibrinogen mediated by α_V_β_3_, and showed little/no activity inhibiting α_4_β_1_-mediated MOLT 4 cell attachment. 

Details of conformational changes in the RGD-tri-peptide within ddADAM-15 while interacting with integrins remain unclear. A putative binding model has been constructed based on the 3D structure of integrin α_V_β_3 _in complex, with a cyclic penta-peptide presenting the RGD sequence [98], where the RGD motif of ddADAM-15 is located at 64–66 aa (R^64^GD^66^) and fits a crevice between the propeller (α subunit) and βA (β_1 _subunit) domains on the β_1_-associated complex headpiece. Conversely, the RGD motif in atragin (538–540 aa according to the sequence number of atragin), located at the end of the η4 helix of the K-like domain, is inaccessible for integrin molecules implying that K-like domains bind to the integrin through a non-RGD region, such as the hypervariable region (HVR) [61,99]. In this review, we have mainly described the RGD-containing D-domain in ADAM-15 interacting with integrins, the non-RGD-containing D-domains in other ADAMs are also reported to associate with integrins, e.g., ADAM-23 can bind to α_V_β_3_[100] and ADAM-28 can interact with α_4_β_1 _[101]. However, the structural basis for these associations has yet to be defined. Therefore, further studies are required to analyze ddADAM-15, ADAM-15 and other ADAMs by X-ray crystallography to gain structural information and increase understanding of ADAM-integrin interaction.
